# Differential polarization and activation dynamics of systemic T helper cell subsets after aneurysmal subarachnoid hemorrhage (SAH) and during post-SAH complications

**DOI:** 10.1038/s41598-021-92873-x

**Published:** 2021-07-09

**Authors:** Shafqat Rasul Chaudhry, Ulf Dietrich Kahlert, Thomas Mehari Kinfe, Elmar Endl, Andreas Dolf, Mika Niemelä, Daniel Hänggi, Sajjad Muhammad

**Affiliations:** 1 Department of Neurosurgery, University Hospital Bonn, University of Bonn, 53127 Bonn, Germany; 2 Shifa College of Pharmaceutical Sciences, Shifa Tameer-e-Millat University, Islamabad, 44000 Pakistan; 3 Department of Neurosurgery, Faculty of Medicine and University Hospital Düsseldorf, Heinrich-Heine University Düsseldorf, 40225 Düsseldorf, Germany; 4 Division of Functional Neurosurgery and Stereotaxy, Friedrich‐Alexander University (FAU) Erlangen‐Nürnberg, 91054 Erlangen, Germany; 5 Flow Cytometry Core Facility, Department of Experimental Immunology, Faculty of Medicine, University Hospital Bonn, University of Bonn, 53127 Bonn, Germany; 6 Department of Neurosurgery, Helsinki University Hospital and University of Helsinki, Helsinki, Finland

**Keywords:** Stroke, CD4-positive T cells

## Abstract

Aneurysmal subarachnoid hemorrhage (SAH) is associated with high morbidity and mortality. Devastating post-SAH complications, such as cerebral vasospasm (CVS), delayed cerebral ischemia or seizures to mention a few, are mainly responsible for the poor clinical outcome. Inflammation plays an indispensable role during early brain injury (EBI) and delayed brain injury (DBI) phases over which these complications arise. T helper cells are the major cytokine secreting cells of adaptive immunity that can polarize to multiple functionally unique sub-populations. Here, we investigate different CD4+ T cell subsets during EBI and DBI phases after SAH, and their dynamics during post-SAH complications. Peripheral venous blood from 15 SAH patients during EBI and DBI phases, was analyzed by multicolour flowcytometry. Different subsets of CD3+ CD4+ T cells were characterized by differential cell surface expression of CXCR3 and CCR6 into Th1, Th2, Th17, whereas Tregs were defined by CD25^hi^CD127^lo^. The analysis of activation states was done by the expression of stable activation markers CD38 and HLA-DR. Interestingly, compared to healthy controls, Tregs were significantly increased during both EBI and DBI phases. Different activation states of Tregs showed differential significant increase during EBI and DBI phases compared to controls. HLA-DR− CD38+ Tregs were significantly increased during DBI phase compared to EBI phase in SAH patients developing CVS, seizures and infections. However, HLA-DR− CD38− Tregs were significantly reduced during EBI phase in patients with cerebral ischemia (CI) compared to those without CI. HLA-DR− CD38− Th2 cells were significantly increased during EBI phase compared to controls. A significant reduction in Th17/Tregs and HLA-DR− CD38+ Th17/Tregs ratios was observed during both EBI and DBI phases compared to controls. While HLA-DR− CD38− Th17/Tregs and HLA-DR− CD38− Th1/Th2 ratios were impaired only during EBI phase compared to controls. In conclusion, CD4+ T cell subsets display dynamic and unique activation patterns after SAH and during the course of the manifestation of post-SAH complications, which may be helpful for the development of precision neurovascular care. However, to claim this, confirmatory studies with larger patient cohorts, ideally from different ethnic backgrounds, are required. Moreover, our descriptive study may be the grounds for subsequent lab endeavors to explore the underlying mechanisms of our observations.

## Introduction

Aneurysmal subarachnoid hemorrhage (SAH) involves a sentinel subarachnoidal bleed as a consequence of a ruptured intracranial aneurysm and confers significant morbidity and mortality among other strokes^1–3^. Despite the treatment of the bleeding aneurysms by endovascular coiling or neurosurgical clipping, still majority of the patients confront life threatening complications including cerebral vasospasm (CVS), delayed cerebral ischemia/infarction (DCI), seizures, cortical spreading depression (CSD), chronic hydrocephalus, infections and deteriorate later on^1,4,5^. The brain injury after SAH occurs in two phases; an early brain injury (EBI) occurs due to elevated intracranial pressure due to ruptured aneurysms within 72 h of SAH and a delayed brain injury due to secondary tissue ischemia over 3–14 days after SAH^6,7^. A great body of evidence highlights the critical role of sterile inflammation during these brain injury phases, which is marked by the release of “damage associated molecular pattern molecules”—DAMPs, cytokines and activation of immune cells^8–13^.

Activated lymphocytes are known to facilitate both the clearance of damaged or infected cells and neutralization of microbes owing to their ability to secrete different cytokines^14^. The involvement of lymphocytes in the pathophysiology of SAH is evidenced by the observations of lymphocyte infiltrations in the arteries affected by CVS, harvested upon autopsies^15^ and in aneurysmal tissue resections obtained during aneurysmal clipping, showing several T cells, but rarely B cells^16^. An experimental SAH study consolidated this observation showing T cell infiltrates in the subarachnoid space, which were closely associated with the cerebral blood vessels^17^. Interestingly, after two days of SAH induction, the authors found that the T cells and CD4+ T cells (T helper cells) achieved the peak levels and persisted significantly in high levels until day 7 compared to sham non-SAH animals^17^. Furthermore, the peripheral T cells were significantly elevated on day 3^17^. Further adding to the evidence of misregulated T-cell biology in the microenvironment of SAH manifestation, T cells expressing IL-1β were also detected in the CSF of SAH patients^18^. Intriguingly, various studies have also evaluated T helper cell response in the peripheral circulation after SAH^14,19–22^. The suppressor lymphocytes (now known as Treg cells) from the peripheral blood of SAH patients were shown to have impaired proliferative capacity^23^, and afterwards, Chrapusta and colleagues have documented a significant decline in CD4+ T cells in SAH patients treated with dexamethasone compared to controls^19^. The decreased cellular proliferation was associated with increased adhesion of T cells and T cell co-stimulatory properties in the peripheral blood of SAH patients^23^. Interestingly, a more aggressive T cell adhesion and co-stimulation profile along with increased CD4+ T cells was evident particularly in SAH patients presenting with CVS^19^. These observations highlight an indispensable role of CD4+ T cells in the post-SAH pathology.

Over the recent years, various subsets of CD4+ T cells have been characterized such as Th1, Th2, Th17 and Treg cells. Th1 cells secrete interferon (IFN)-γ, express T-bet signature transcription factor and induce cell mediated immune responses against intracellular pathogens^14,19–22^. Th2 cells secrete IL-4, IL-5 and IL-13 as major cytokines, express GATA3 and mediate antiparasitic immunity and allergic responses^24^. Th17 cells secrete an important pro-inflammatory cytokine IL-17, express RORγt and provide protection against bacterial and fungal infections through the recruitment of neutrophils; and are also implicated in autoimmune diseases^25,26^. Treg cells express FOXP3 and release cytokines such as IL-10, TGF-β, and IL-35^27^. These cells play a critical role in immune tolerance and prevention against autoimmune diseases by inhibiting the activity of all immune cells^27^.

Interestingly, SAH has been shown to lead to lymphopenia^20,21^, however, T helper cells expressing transient activation marker CD69 have been shown to increase after SAH^20^. Contrarily, an early significant decrease in T helper cells has been documented in SAH patients with acute focal neurological deficits and may explain the immunosuppression after SAH^21^. Recently, a study found decrease in T helper cells and Treg cells in SAH patients undergoing neurosurgical microclip obliteration of their aneurysms and developing fever^14^. Overall, however, a very limited number of studies have been done so far aiming to investigate the response of various subsets of CD4+ T cells after SAH. For instance, in an experimental endovascular puncture model of SAH, statins administration was found to polarize Th1 cells into Th2 cells^28^. Further, a clinical study comprising both ruptured and unruptured intracranial aneurysms has shown a significant increase in Th17 cells and decrease in Th2 cells compared to controls^22^. However, studies aiming to investigate the dynamics of different CD4+ T cell subsets and their activation states after SAH in detail have not been performed so far. Therefore, the current study aims to fill this gap of knowledge by characterizing the detailed dynamics of CD4+ T cell subtypes during early and delayed brain injury phases after SAH- and during post-SAH complications.

## Methods

### Ethics statement

This study was performed according to the guidelines of the Helsinki declaration and was approved by the local ethics committee of the medical faculty of the University of Bonn, Germany (Reference Number: LfD 138/2011). Informed consent was obtained from all subjects (SAH patients and healthy controls) or from patients’ legal guardians for unconscious SAH patients by the treating neurosurgeon.

### Patient population

We included 15 SAH patients prospectively and 10 healthy controls in this study. Peripheral blood was planned between days 1 and 3 (denoting Early Brain Injury (EBI) phase) and between days 7 and 9 (denoting Delayed Brain Injury (DBI) phase). Blood from healthy patients was taken at one time point. Indeed, except a few patients, first sample was collected within 24 h of hospital admission and second sample was collected on day 7 in the majority of SAH patients. The exclusion and inclusion criteria, patient monitoring and treatment, and description of different complications have been described elsewhere^8,10,12^. The detailed characters of patient population and controls are represented in Table 1.

**Table 1:** Characters of healthy controls and SAH patients.

**﻿Controls (n)**	10
Age (years) (mean ± SD)	34.62 (± 13.24)
Females (n)	06
**﻿SAH (n)**	15
Age (years) (mean ± SD)	52.88 (± 11.81)
Females (n)	06
**Treatment modality**	
Neurosurgical clipping (n)	06
Endovascular coiling (n)	09
Intraventricular hemorrhage: IVH (n)	04
Intracerebral bleeding: ICB (n)	06
Both ICB and IVH (n)	04
**Hunt and Hess grade (median)**	4
Good grade I–III (n)	06
Poor grade IV–V (n)	09
**Fischer grade (median)**	3
1 (n)	0
2 (n)	0
3 (n)	12
4 (n)	03
CVS (n)	09
Cerebral ischemia (n)	10
Seizures (n)	09
VP-shunt dependent hydrocephalus (n)	05
Infections (n)	08
Delayed Ischemic Neurological Deficits: DINDs (n)	03
**Aneurysm location**	
Anterior circulation (n)	13
Posterior circulation (n)	02
**GOS (median)**	3
Poor outcome 1–3 (n)	09
Good outcome 4–5 (n)	06
**mRS (median)**	4
Good outcome 0–2 (n)	06
Poor outcome 3–6 (n)	09

Different subsets of CD4^+^ T cells and Tregs were investigated by the approach described by Maecker, et al.^29^.

### Collection of the blood sample and preparation for staining

The peripheral blood of the SAH patients and controls was retrieved in EDTA blood tubes (S-Monovette^®^ Sarstedt, Germany). Erythrocyte lysis was achieved with erythrocyte lysis buffer (eBioscience, Germany) at room temperature. Afterwards, centrifugation of the cells was carried out at 350×*g* for 5 min at 4 °C. A volume of 2 mL of an ice cold FACS flow cytometry buffer (BD Biosciences, Germany) was used to wash the cells after discarding the supernatant. Cells were resuspended in 1 mL of FACS buffer after washing and then, were counted using countess cell counting slides (Catalog # C10283, Eugene, Oregon, USA) by using Countess™ automated cell counter (ThermoFischer scientific, Germany). The final concentration of cells was adjusted to one million cells per 100 µL with the FACS buffer. Next, flow cytometry tubes (5 mL; Catalog # 55.1578, Sarstedt, Germany) were labelled for stained cells and fluorescence minus one (FMO) controls and 100 µL aliquots of the cells were dispensed into the respective tubes. A drop of Ultracomp eBeads (eBioscience, Germany) was dispensed into each tube containing 100 µL of FCS buffer for the acquisition of the single stained compensation controls. Next, Human Fc block pure (Catalog # 564220, BD Biosciences, Germany) was added to the cells and the cells were further incubated for 10 min on ice^30^.

### Staining of blood cells

The cells were then stained with a panel of anti-human antibodies conjugated with different fluorophores against various T cell markers and consisted of CD45 APC-H7, CD3 PE-Cy7, CD4 BV605, CXCR3 APC, CCR6 BUV737, CD25 PerCP-Cy5.5, CD127 FITC, HLA-DR BV421 and CD38 PE (BD Biosciences, USA). Supplementary Table S1 represents the respective clones and catalog details of these antibodies. A sequential staining of cells was performed for chemokine receptors (CXCR3, CCR6) at room temperature giving an interval of at least 5 min before addition of the next antibody as proposed by Jalbert et al.^31^. Next, the cells in the stained sample and the FMO control tubes were stained with a master mix of antibodies prepared in Brilliant Violet Staining buffer (BV Buffer, BD Biosciences, Germany) for twenty minutes on ice. Later on, 2 mL of the FACS buffer was added to wash the cells and subsequently the cells were resuspended in approximately 500 µL of the FACS buffer. Hoechst 33258 dye (Sigma Aldrich, Germany), a water soluble fluorescent dye, was added (0.1 µg/10 µL) approximately one minute before acquisition of each tube to discriminate between live and dead cells^30^.

### Acquisition and analysis of CD4^+^ T cell subsets

The cells were then analyzed on LSR Fortessa™ cell analyzer (BD Biosciences, CA, USA) at the Flow Cytometry Core Facility at the Institute of Experimental Immunology, University of Bonn. The flow cytometer settings were validated using 8 peaks SPHERO™ Calibration Particles as an internal reference (Catalog # 559123, BD Biosciences, CA, USA) and instrument performance was monitored every day by running CS&T beads (Catalog # 655050, BD Biosciences, CA, USA) according to the vendors recommendations. For both panels, around 350–400 thousand CD45+ leucocyte events were acquired for stained cells and 100 thousand CD45+ events for FMO controls. About 10,000 all events were acquired for single stained compensation controls. Compensation controls were stained and measured freshly, on each day the analysis was performed. The gating strategy is given in Fig. S2. BD FACSDiva™ v6.2 for windows 7 (BD Biosciences, USA) software was used during acquisition on LSR Fortessa, while the data was analyzed afterwards using FlowJo^TM^ Software version 10.2 for Microsoft Windows 7 (Treestar, Ashland, OR)^30^.

### Statistical analysis

The normality of the data was assessed by Shapiro–Wilk test or Kolmogrov–Smirnov test. Normally distributed data was expressed as mean ± SEM, whereas non-normally distributed data was displayed using box plots with whiskers representing median, interquartile range and minimum and maximum values. One way analysis of variance (ANOVA) followed by Tukey’s/Bonferroni's multiple comparisons post hoc test was used for comparison between controls and SAH patients during both EBI and DBI phases for normally distributed data. For non-normally distributed data, Krukal–Wallis test was used followed by Dunn’s multiple comparisons post hoc test. A *p* value less than 0.05 was considered as a significant difference between the groups being compared. The data was analyzed by using GraphPad Prism 5.00 for windows (CA, USA).

## Results

### CD4+ T cells response after SAH

**Figure 1:**
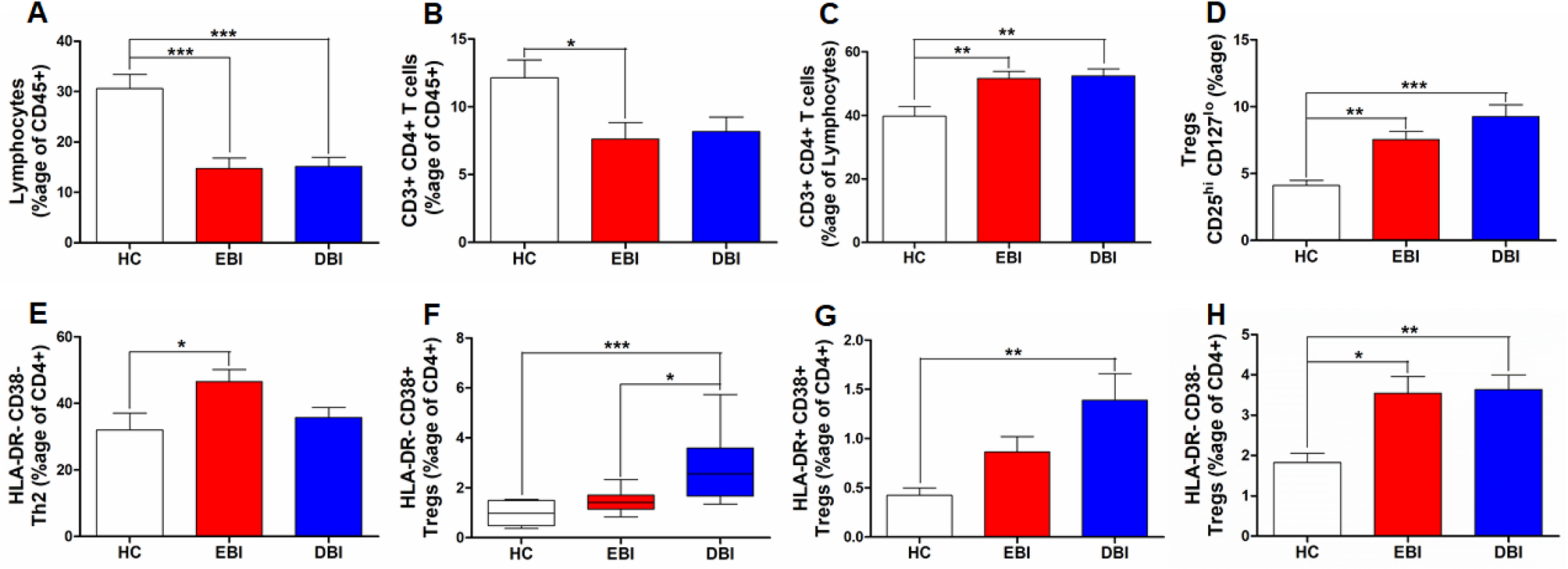
Comparison of peripheral blood: (**A**) lymphocytes (low SSC and high CD45 expressing cells) after SAH during EBI and DBI phases with healthy controls; (**B**) CD4+ T cells (expressed as %age of CD45 + leukocytes) after SAH during EBI and DBI phases with healthy controls; (**C**) CD4+ T cells (expressed as %age of lymphocytes) after SAH during EBI and DBI phases with healthy controls; (**D**) Tregs (CD25^hi^ CD127^lo^, expressed as %age of CD3+ CD4+ T cells) after SAH during EBI and DBI phases with healthy controls; (**E**) HLA-DR− CD38− Th2 cells (expressed as %age of CD3+ CD4+ T cells) after SAH during EBI and DBI phases with healthy controls; (**F**) HLA-DR− CD38+ Tregs (expressed as %age of CD3+ CD4+ T cells) after SAH during EBI and DBI phases with healthy controls; (**G**) HLA-DR+ CD38+ Tregs (expressed as %age of CD3+ CD4+ T cells) after SAH compared to healthy controls during EBI and DBI phases, (**H**) HLA-DR− CD38− Tregs (expressed as %age of CD3+ CD4+ T cells) after SAH during EBI and DBI phases compared to healthy controls. One way ANOVA followed by Tukey’s multiple comparisons test for normally distributed data. Kruskal Wallis test followed by Dunn’s multiple comparisons test for non-normally distributed data; A *p* value < 0.05 was considered as a significant difference. *Indicates a *p* value < 0.05, **indicates a *p* value < 0.01, ***indicates a *p* value < 0.001. *HC* healthy controls (n = 10), *EBI* early brain injury phase after SAH covering days 1–3 (n = 15), *DBI* delayed brain injury phase after SAH covering days 7–9 (n = 15).

Lymphocytes in the peripheral blood of SAH patients and healthy controls were identified by their low SSC and high CD45 expression and were expressed as percentage of CD45+ cells to reflect changes compared to all the leukocytes. A lymphopenic response was observed in SAH patients during both EBI and DBI phases compared to healthy controls (Fig. 1A). Taking into account this lymphopenic response, CD4+ T cells were also significantly reduced during EBI phase among all the leukocytes, i.e., when expressed as percentage of CD45+ leukocytes (Fig. 1B). However, no significant difference was observed during DBI phase (Fig. 1B). Interestingly, CD4+ T cells were significantly increased among the lymphocyte population during both EBI and DBI phases compared to the controls (Fig. 1C).

### Regulatory T cells (Tregs) were elevated after SAH

T helper cells have been categorized based upon the expression of signature transcription factors and cytokine profiles into various subtypes such as Th1, Th2, Th17 and Treg cells^24,25,27^. After assessing CD4+ T cells response, we investigated the frequency of Tregs (CD25^hi^ CD127^lo^) and different subsets of CD4+ T cells (Th1, Th2 and Th17) based on differential cell surface expression of chemokine receptors CXCR3 and CCR6 as described by Maecker, et al.^29^. Apparently, the levels of Th1 cells (CXCR3+ CCR6−) and Th17 cells (CXCR3− CCR6+) appear to be low during EBI phase, however, there was no statistically significant difference compared to controls during both EBI and DBI phases (Fig. S1A,B). Likewise, there was also no significant difference in Th2 cells (CXCR3− CCR6−) during both EBI and DBI phases compared to healthy controls (Fig. S1C). Intriguingly, Treg cells (CD25^hi^ CD127^lo^), which are immunosuppressive cells, were significantly increased during both EBI and DBI phases compared to controls (Fig. 1D).

### Assessment of activation states of CD4+ T cell subsets and Tregs

Activation markers can provide insights into a disease progress and activity, and therefore, could have a biomarker potential^29^. In opposition to CD69, which is transiently expressed, CD38 and HLA-DR represent permanent activation markers^29^. We next investigated the activation markers HLA-DR and CD38 on these different CD4+ T cell subsets. A significant increase in Th2 cells lacking both of these activation markers (HLA-DR− CD38−) was observed during EBI compared to healthy controls, however, during DBI phase the levels of these cells declined, leaving no significant difference compared to controls (Fig. 1E). HLA-DR− CD38+ Tregs were significantly increased during DBI phase compared to controls as well as EBI phase (Fig. 1F). Tregs expressing both activation markers (CD38+ HLA-DR+) were significantly increased during DBI phase compared to controls (Fig. 1G). However, Tregs lacking both of these activation markers (HLA-DR− CD38−) were significantly increased during both EBI and DBI phases compared to controls like their parent population (Fig. 1H).

### T helper cells, their activation states and post-SAH complications

**Figure 2:**
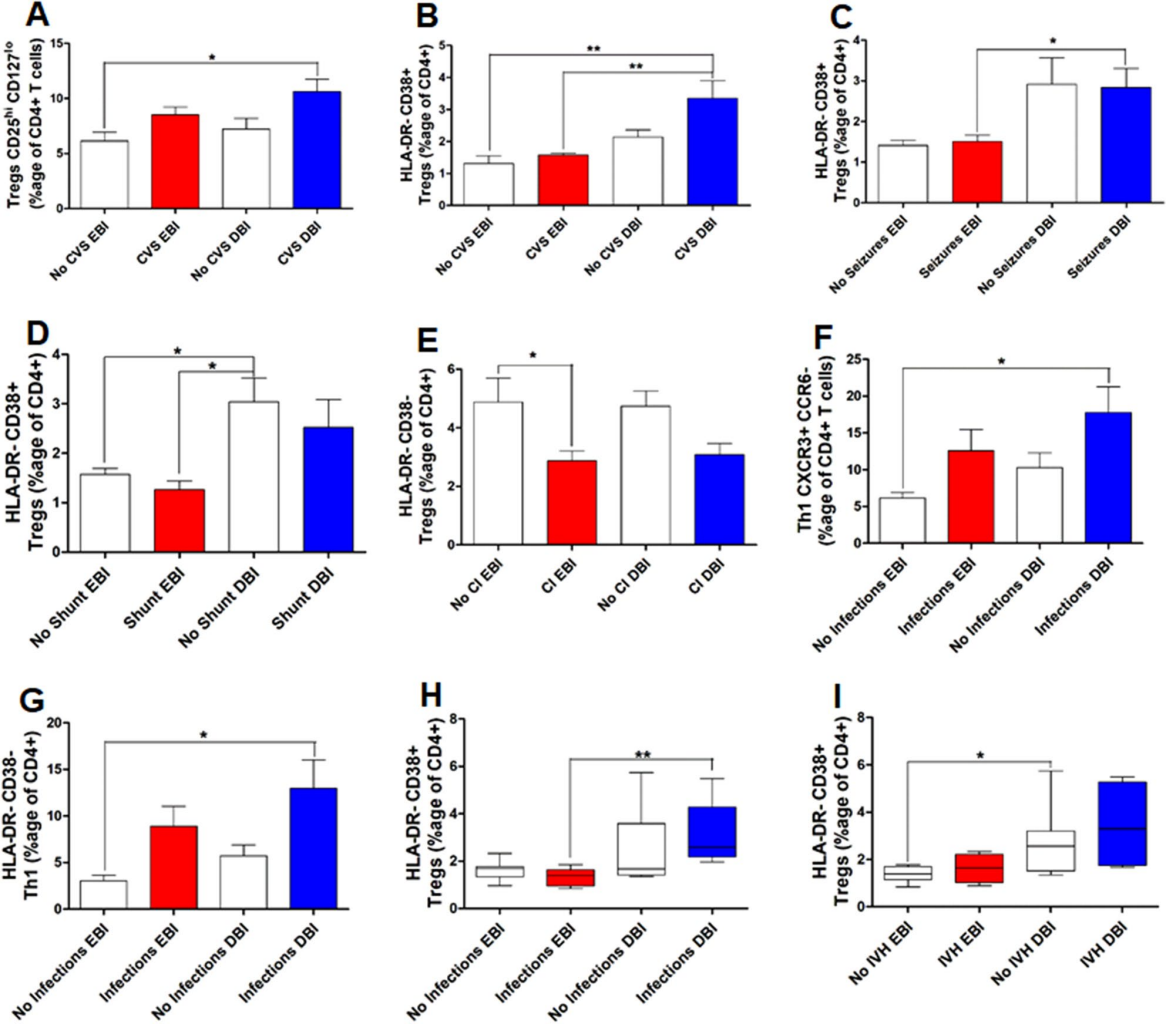
Comparison of peripheral blood: (**A**) Tregs (CD25^hi^ CD127^lo^, expressed as %age of CD3+ CD4+ T cells) after SAH in patients with cerebral vasospasm (CVS) and without CVS during EBI and DBI phases; (**B**) HLA-DR− CD38+ Tregs (expressed as %age of CD3+ CD4+ T cells) after SAH in patients with CVS and with no CVS during EBI and DBI phases; (**C**) HLA-DR− CD38+ Tregs (expressed as %age of CD3+ CD4+ T cells) after SAH in patients with seizures and with no seizures during EBI and DBI phases; (**D**) HLA-DR− CD38+ Tregs (expressed as %age of CD3+ CD4+ T cells) after SAH in patients with shunt dependent chronic hydrocephalus and with no shunt dependent chronic hydrocephalus during EBI and DBI phases; (**E**) HLA-DR− CD38− Tregs (expressed as %age of CD3+ CD4+ T cells) after SAH in patients with cerebral ischemia/infarction (CI) and with no CI during EBI and DBI phases; (**F**) Th1 cells (CXCR3+ CCR6− , expressed as %age of CD3+ CD4+ T cells) after SAH in patients with infections and without infections during EBI and DBI phases; (**G**) HLA-DR− CD38− Th1 cells (expressed as %age of CD3+ CD4+ T cells) after SAH in patients with infections and without infections during EBI and DBI phases; (**H**) HLA-DR− CD38+ Tregs (expressed as %age of CD3+ CD4+ T cells) after SAH in patients with infections and without infections during EBI and DBI phases; (**I**) HLA-DR− CD38+ Tregs (expressed as %age of CD3+ CD4+ T cells) after SAH in patients with IVH and without IVH during EBI and DBI phases. One way ANOVA followed by Tukey’s/Bonferroni's multiple comparisons test for normally distributed data. Kruskal Wallis test followed by Dunn’s multiple comparisons test for non-normally distributed data; A *p* value < 0.05 was considered as a significant difference. *Indicates a *p* value < 0.05, **indicates a *p* value < 0.01, ***indicates a *p* value < 0.001. *EBI* early brain injury phase after SAH covering days 1–3 (n = 15), *DBI* delayed brain injury phase after SAH covering days 7–9 (n = 15).

SAH patients confront with various deteriorating complications during EBI and DBI phases leading to poor clinical outcomes^1,2,4,5^. We next investigated the response of T helper cell subsets and their activation states in various post-SAH complications such as CVS, seizures, chronic hydrocephalus, cerebral ischemia (CI) and infections. One of the most feared and frequent complication of SAH is CVS^1^. There was no significant difference in Tregs in SAH patients developing CVS and those developing no CVS during EBI and DBI phases (Fig. 2A). However, Tregs were significantly increased in SAH patients with CVS during DBI phase compared to the SAH patients without CVS during EBI phase (Fig. 2A). Similar to their parent population, HLA-DR− CD38+ Tregs were significantly increased in SAH patients with CVS during DBI phase compared to SAH patients without CVS during EBI phase (Fig. 2B). Interestingly, HLA-DR− CD38+ Tregs were significantly increased in SAH patients with CVS during DBI phase compared to EBI phase (Fig. 2B). SAH patients who developed seizures also did not show any significant difference compared to those without seizures (Fig. 2C). However, HLA-DR− CD38+ Tregs were significantly increased in SAH patients developing seizures during DBI phase compared to EBI phase (Fig. 2C). Several SAH patients develop ventriculoperitoneal shunt dependent chronic hydrocephalus after SAH. In our study group, SAH patients developing chronic hydrocephalus did not show any significant difference in HLA-DR− CD38+ Tregs compared to those developing no chronic hydrocephalus during both EBI and DBI phases (Fig. 2D). However, SAH patients who did not develop chronic hydrocephalus have significantly increased levels of HLA-DR− CD38+ Tregs during DBI phase compared to EBI phase (Fig. 2D). Further, SAH patients who did not develop chronic hydrocephalus have significantly increased levels of HLA-DR− CD38+ during DBI phase compared to SAH patients who developed chronic hydrocephalus during EBI phase (Fig. 2D). SAH patients who developed cerebral ischemia/infarction (CI) showed a significant decrease in Tregs during EBI phase compared to SAH patients who did not develop CI during DBI phase (data not shown). Interestingly, SAH patients who developed CI showed a significant reduction in Tregs lacking both activation markers (HLA-DR− CD38−) compared to those who did not develop CI during EBI phase (Fig. 2E). However, no significant difference was observed during DBI phase in HLA-DR− CD38− Tregs in SAH patients with CI compared to SAH patients without CI (Fig. 2E). Another factor that contributes to the poor clinical outcome is the frequent development of infections in SAH patients^32^. SAH patients who developed infections did not show any significant difference in Th1 (CXCR3+ CCR6−) and HLA-DR− CD38− Th1 cells compared to SAH patients without infections during both EBI and DBI phases (Fig. 2F,G). However, both Th1 cells and HLA-DR﻿− CD38− Th1 cells displayed significant increase in SAH patients with infections during the DBI phase compared to SAH patients without infections during EBI phase (Fig. 2F,G). Further during DBI phase, HLA-DR− CD38+ Tregs were significantly elevated in SAH patients contracting different infections compared to SAH patients without infections during EBI phase (Fig. 2H). Some SAH patients also present with extravasation of the blood into the cerebral ventricles^1^. SAH patients presenting with IVH did not show any significant difference compared to SAH patients without IVH in HLA-DR− CD38+ Tregs (Fig. 2I) during both EBI and DBI phases. But, surprisingly this population of Tregs (HLA-DR− CD38+) showed significant increase during DBI phase compared to EBI phase in only SAH patients without IVH (Fig. 2I).

### Imbalance of Th1/Th2, Th17/Tregs and their activation states after SAH

**Figure 3:**
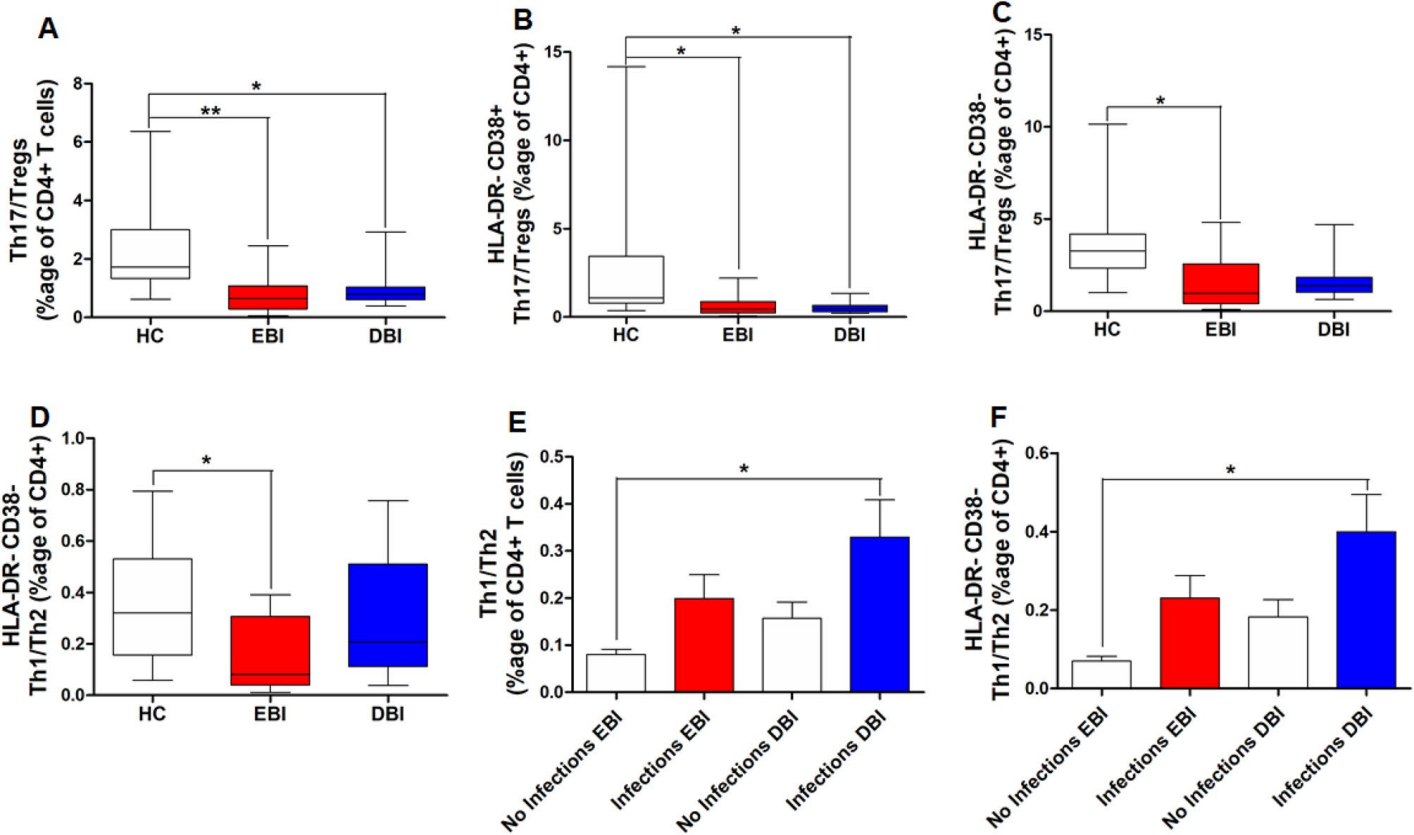
Comparison of peripheral blood: (**A**) Th17/Tregs ratio after SAH during EBI and DBI phases with healthy controls; (**B**) HLA-DR− CD38+ Th17/Tregs ratio after SAH during EBI and DBI phases with healthy controls; (**C**) HLA-DR− CD38− Th17/Tregs ratio after SAH during EBI and DBI phases with healthy controls; (**D**) HLA-DR− CD38− Th1/Th2 cells ratio after SAH during EBI and DBI phases with healthy controls; (**E**) Th1/Th2 cells ratio after SAH in patients with infections and without infections during EBI and DBI phases; (**F**) HLA-DR− CD38− Th1/Th2 cells ratio (expressed as %age of CD3+ CD4+ T cells) after SAH in patients with infections and without infections during EBI and DBI phases. One way ANOVA followed by Tukey’s multiple comparisons test for normally distributed data. Kruskal Wallis test followed by Dunn’s multiple comparisons test for non-normally distributed data; A *p* value < 0.05 was considered as a significant difference. *Indicates a *p* value < 0.05, **indicates a *p* value < 0.01, ***indicates a *p* value < 0.001. *HC* healthy controls (n = 10), *EBI* early brain injury phase after SAH covering days 1–3 (n = 15), *DBI* delayed brain injury phase after SAH covering days 7–9 (n = 15).

Several studies describe the imbalance of T helper cells by assessing the ratio of Th1/Th2 and Th17/Tregs to delineate the pathological changes. We have also assessed the Th1/Th2 and Th17/Tregs ratios in SAH patients and controls. There was no significant difference in Th1/Th2 ratio among controls and SAH patients (data not shown). Intriguingly, Th17/Tregs ratio was significantly lower in SAH patients during both EBI and DBI phases compared to controls (Fig. 3A). HLA-DR﻿− CD38+ Th17/Tregs ratio was also significantly lower during EBI and DBI phases in SAH patients compared to controls (Fig. 3B). SAH patients also displayed significantly lower ratios of HLA-DR﻿− CD38− Th17/Tregs and HLA-DR− CD38− Th1/Th2 during EBI phase compared to controls (Fig. 3C,D). Similar analysis in SAH patients developing different complications revealed significant changes only in SAH patients contracting different infections. SAH patients contracting different infections displayed higher Th1/Th2 and HLA-DR﻿− CD38− Th1/Th2 ratios during DBI phase compared to SAH patients without infections during EBI phase (Fig. 3E,F).

### Correlations of different T helper cell subsets and their activation states with different post-SAH complications

Different subsets of T helper cells showed some significant correlations during EBI and DBI phases with various post-SAH complications as shown in Table 2. Interestingly, Tregs were positively correlated with the development of CVS and negatively correlated with CI development in SAH patients during EBI phase. HLA-DR﻿− CD38− Tregs were also negatively correlated with CI development in SAH patients during both EBI and DBI phases. However, HLA-DR+ CD38+ Tregs were positively correlated with the contraction of infections during DBI phase. HLA-DR− CD38+ Tregs were negatively correlated with the development of delayed ischemic neurological deficits (DINDs) during DBI phase. HLA-DR+ CD38+ Th1 cells were positively correlated with the development of seizures during EBI phase and HLA-DR+ CD38− Th1 cells were negatively correlated with ICB in SAH patients during DBI phase. HLA-DR− CD38+ Th2 cells were positively correlated with CI development during DBI phase.

**Table 2:** Correlations of different T helper cell subsets and their activation states with different post-SAH complications.

Sr. #	T cell subsets	Post-SAH injury phase	Post-SAH characters and complications	Spearman Rank correlation	*p* value
1	Tregs	EBI	CVS	0.535	0.040
2	Tregs	EBI	CI	− 0.524	0.045
3	Tregs HLA-DR− CD38+	DBI	DINDs	− 0.541	0.037
4	Tregs HLA-DR− CD38−	EBI	CI	− 0.556	0.031
5	Tregs HLA-DR− CD38−	DBI	CI	− 0.556	0.031
6	Tregs HLA-DR+ CD38+	DBI	Infections	0.526	0.044
7	Th1 HLA-DR+ CD38+	EBI	Seizures	0.520	0.047
8	Th1 HLA-DR+ CD38−	DBI	ICB	− 0.521	0.047
9	Th2 HLA-DR− CD38+	DBI	CI	0.524	0.045

## Discussion

Rapid implementable, minimal invasive diagnostics, that more dynamically and sensitively monitor neurovascular disease progression are highly needed. We utilized peripheral blood immune cell quantification and characterization to propose a concept to serve this purpose. Our work complements the closure of lack of knowledge, as very limited number of studies have investigated the major T helper cell subsets after this potentially lethal disease—SAH^19–21^. In our explorative observational clinical study, we have characterized different CD4+ T cell subsets based on differential expression of cell surface receptors along with stable activation markers during EBI and DBI phases after SAH.

Immunosuppression is observed after SAH^32^. Several lines of evidence indicate a lymphopenic response after SAH^20,21^. In line with these findings, we found a significant decrease in lymphocytes and CD4+ T cells (expressed as frequencies of CD45+ leukocytes to reflect their changes among all other leukocytes) after SAH compared to controls (Fig. 1A,B). However, CD4+ T cells among lymphocyte population were increased during both EBI and DBI phases after SAH as compared to controls (Fig. 1C), which is in par with findings from Moraes et al.^20^. Strikingly, among the T helper cell subtypes, Tregs and various activated states of Tregs were significantly increased during both EBI and DBI phases after SAH (Fig. 1D,F–H). The robust increase in Tregs after SAH in our study is in accordance with that observed after intracerebral hemorrhage^33^. HLA-DR− CD38− Th2 cells showed significant increase during EBI phase only compared to controls (Fig. 1E). The clinical outcome of SAH patients is worsened by complications that develop over two weeks after the sentinel bleed, even though the ruptured aneurysms are successfully secured^34^. Therefore, we have analyzed the dynamics of CD4+ T cell subsets during different post-SAH complications. Interestingly, various activated states of Tregs showed differential expression during both EBI and DBI phases in SAH patients with CVS, seizures, infections and shunt-dependent chronic hydrocephalus (Fig. 2). Approximately, one third of SAH patients suffer from seizures^5^ and two thirds develop cerebral vasospasm, which is the most feared and frequent complication after SAH^1^. Consequently, the dysregulation of HLA-DR− CD38+ Tregs may be a promising biomarker in context to these post-SAH complications. Interestingly, we found an impairment in the balance of Th17/Tregs during EBI and DBI phases after SAH compared to controls (Fig. 3A), which is in line with a recent study involving severe grade SAH patients^35^. Likewise, various activation states of Th17/Tregs showed similar impairment during EBI and DBI phases after SAH compared to controls (Fig. 3B,C). Th1/Th2 cells balance and their respective activation states also showed impairment during EBI phase after SAH and in SAH patients with infections during DBI phase (Fig. 3D–F). Our data calls for confirmation studies to validate the Th17/Treg impairment during SAH complications.

Another peculiar aspect of our study of helper T cell subsets was the analysis of fresh samples immediately after collection from the patients and the inclusion of permanent cell surface activation markers such as CD38 and HLA-DR as opposed to transient activation marker CD69^29^. These activation markers of T cells may serve as the biomarkers of disease activity and severity and may provide important insights into the disease prognosis or underlying immunological dysregulation contributing to the disease^29^. Analysis of these activation states of T helper cells showed that both CD38 and HLA-DR are differentially expressed on these different subtypes of T helper cells (Figs. 1, 2, 3). T cells that show the expression of CD38 have been found to be large in size, contain more granules, show reduced proliferation and increased cytokine secretion^36^. T cells expressing both HLA-DR and CD38 signify the activated states and indicate the advancement of the disease process in case of various inflammatory diseases^37^. The population of Treg cells that express HLA-DR represent terminally activated effector Treg cells, which have acquired the ability to profoundly inhibit the proliferation of conventional T cells and readily produce cytokines in comparison to Tregs lacking HLA-DR expression^27^. The current study shows various novel findings with respect to the expression of these activation markers, which need validation in larger observational studies.

Despite all of the above-described interesting findings, our study has several fundamental limitations, which need to be acknowledged when critically assessing our stated hypothesis. The major limitation is the comparatively small sample size. The authors acknowledge that the overall relatively rare clinical occurrence of the disease contributes to this fact, however, confirmatory study that includes patients from different backgrounds, with sampling performed in multiple different treatment centers is warranted. Although our quality control measures aim to standardize the procedures as much as possible^38^, however, we cannot exclude that logistics from patient bedside to the laboratory (transport time, chilling conditions during transport etc.) as well as time to staining and FACS data acquisition varied and that is known to risk the introduction of variances on the parameters analyzed. Another limitation of our study is that the absolute numbers of the analyzed immune cell populations were not assessed. Further, our study lacks the information on naïve, effector, effector memory and central memory discrimination of T helper cells along with the assessment of other lymphocyte populations. Moreover, sex and age matching of our study population with control cohort was not possible due to the low number of total patients enrolled in the trial. However, our study advocates the involvement and unveils the complexity of CD4+ T cells response during early and delayed brain injury after SAH and during post-SAH complications. Technically, this study also provides novel insights into high dimensional flow cytometry data in association with clinical parameters of SAH patients as an example of forward-thinking, reverse translational neurosurgical research to instruct subsequent experimental trials for mechanistic exploitation are also warranted.

## Conclusion

CD4+ T cell subsets display dynamic changes during EBI and DBI phases after SAH. CD4+ T cell subsets and their activation states were differentially expressed in specific post-SAH complications. Th17/Tregs ratio and ratios of their activated states were impaired during EBI and DBI phases after SAH. Our data indicates the great potential of peripheral T helper cell-based quantification and characterization for monitoring the disease progression after SAH by minimal invasive means. Given the lack of adequate alternatives to effectively do so, and the ability to monitor rapid changes using flow cytometry, we believe our results pave the scientific grounds justifying the larger follow-up studies dedicated to this issue.

## Supplementary Information

Supplementary Information.
