# Supplementary material for: Differential polarization and activation dynamics of systemic T helper cell subsets after aneurysmal subarachnoid hemorrhage (SAH) and during post-SAH complications
Source: Sci Rep. 2021 Jul 9;11:14226. doi: 10.1038/s41598-021-92873-x (PMC8270974; doi:10.1038/s41598-021-92873-x)
Supplement: Supplementary file 1 — Supplementary Information. [file 41598_2021_92873_MOESM1_ESM.pdf]

**Differential polarization and activation dynamics of systemic T helper cell subsets after aneurysmal subarachnoid hemorrhage (SAH) and during post-SAH complications**

Shafqat Rasul Chaudhry[1,2], Ulf Dietrich Kahlert[3], Thomas Mehari Kinfe[4], Elmar Endl[5], Andreas Dolf[5], Mika Niemelä[6], Daniel Hänggi[3], Sajjad Muhammad[1,3,6]*

[1]Department of Neurosurgery, University Hospital Bonn, University of Bonn, D-53127 Bonn, Germany

[2]Shifa College of Pharmaceutical Sciences, Shifa Tameer-e-Millat University, 44000 Islamabad, Pakistan

[3]Department of Neurosurgery, Faculty of Medicine and University Hospital Düsseldorf, Heinrich-Heine University of Düsseldorf, D-40225 Düsseldorf, Germany

[4]Division of Functional Neurosurgery and Stereotaxy, Friedrich-Alexander University (FAU) Erlangen-Nürnberg, 91054 Erlangen, Germany

[5]Flow Cytometry Core Facility, Department of Experimental Immunology, Faculty of Medicine, University Hospital Bonn, University of Bonn, D-53127 Bonn, Germany

[6]Department of Neurosurgery, Helsinki University Hospital and University of Helsinki, Helsinki, Finland

*Correspondence: Email ID: sajjad.muhammad@med.uni-duesseldorf.de; Tel.: +004915168460755

## Supplementary Figures and Table

**Figure S1.**

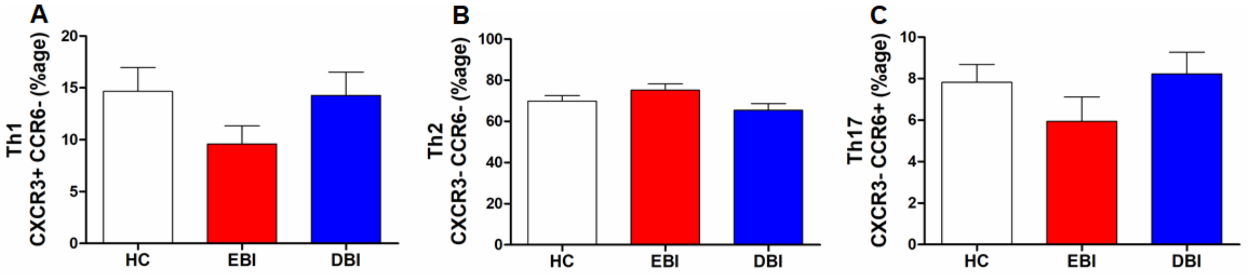


**Figure S1.** Comparison of peripheral blood: (**A)** Th1 (CXCR3+ CCR6-; expressed as %age of CD3+ CD4+ T cells) after SAH with healthy controls during EBI and DBI phases; (**B)** Th2 (CXCR3- CCR6-; expressed as %age of CD3+ CD4+ T cells) after SAH with healthy controls during EBI and DBI phases; (**C)** Th17 (CXCR3- CCR6+; expressed as %age of CD3+ CD4+ T cells) after SAH with healthy controls during EBI and DBI phases. One way ANOVA followed by Tukey's multiple comparisons test for normally distributed data. Kruskal Wallis test followed by Dunn's multiple comparisons test for non-normally distributed data; A $p$ value <0.05 was considered as a significant difference. * indicates a $p$ value <0.05, ** indicates a $p$ value <0.01, *** indicates a $p$ value <0.001. *HC* = Healthy controls (n = 10), *EBI* = Early Brain Injury phase after SAH covering days 1 – 3 (n = 15), *DBI* = Delayed Brain Injury phase after SAH covering days 7 – 9 (n = 15).

**Figure S2. Gating strategy for CD4+ T cell subsets and Treg cells**

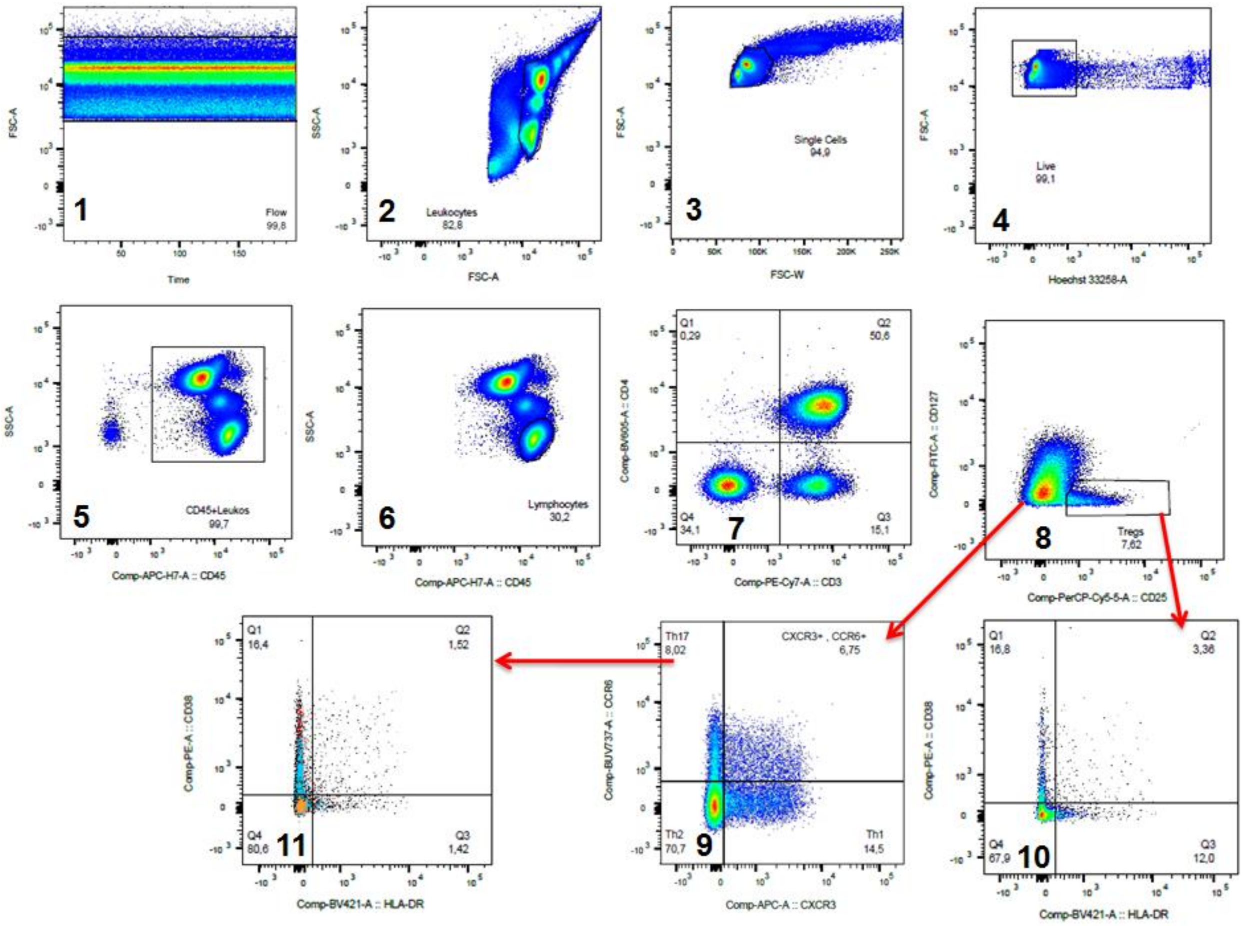


**Figure S2.** The initial gate was made on the events with consistent flow by bivariate plot of Forward Scatter (FSC) and time **(1)**. Then, these events were shown as FSC and side scatter (SSC) plot and a global gate was used to select leucocytes excluding erythrocytes, platelets and debris **(2)**. Next doublets were excluded based on FSC-A vs FSC-W **(3)**. Subsequently, dead cells were excluded based on their Hoechst 33258 staining **(4)**. Then, a bivariate plot of SSC vs CD45 was used to select the CD45+ leucocytes **(5).** Lymphocytes were selected from these CD45+ events by their low side scatter and high CD45 expression **(6)**. These lymphocytes were then displayed as CD3 vs CD4 plot and a quadrant gate was applied **(7)**. Then, from this quadrant gate CD3+ CD4+ events were selected as CD4+ T cells. CD4+ T cells were then displayed on a CD25 vs CD127 plot and $CD25^{hi}CD127^{lo}$ events were gated as Tregs and this gating strategy was based on CD25 FMO control **(8)**. Then, by applying a NOT gating function, events other than Tregs were selected and were named as Th cells. These Th cells were further displayed on a bivariate plot of CXCR3 vs CCR6 **(9)**. Then, a quadrant gate was used based on FMO controls for CXCR3 and CCR6 to identify CXCR3+ CCR6- cells as Th1, CXCR3- CCR6- cells as Th2 and CXCR3- CCR6+ Th17 cells **(9)**. Then, HLA-DR+ and CD38+ cells were identified among these subsets such as Tregs **(10)**, Th1, Th2, and Th17 cells **(11)** by using FMO controls and applying a quadrant gate.

**Table S1. List of antibodies along with their catalog numbers and clones**

| Sr. # | Product | Clone | Catalog no. | Company |
|---|---|---|---|---|
| 1 | Anti-Human CD127 FITC | HIL-7R-M21 | 560549 | Pharmingen$^{TM}$ BD Biosciences |
| 2 | Anti-Human CD183 (CXCR3) APC | 1C6 | 550967 | Pharmingen$^{TM}$ BD Biosciences |
| 3 | Anti-Human CD196 (CCR6) BUV737 | 11A9 | 564377 | Horizon$^{TM}$ BD Biosciences |
| 4 | Anti-Human CD25 PerCP-Cy5.5 | M-A251 | 560503 | Pharmingen$^{TM}$ BD Biosciences |
| 5 | Anti-Human HLA-DR BV421 | G46-6 | 562804 | Horizon$^{TM}$ BD Biosciences |
| 6 | Anti-Human CD4 BV605 | RPA-T4 | 562658 | Horizon$^{TM}$ BD Biosciences |
| 7 | Anti-Human CD3 PE-Cy7 | UCHT1 | 563423 | Pharmingen$^{TM}$ BD Biosciences |
| 8 | Anti-Human CD45 APC-H7 | 2D1 | 560178 | Pharmingen$^{TM}$ BD Biosciences |
| 9 | Anti-Human CD38 PE | HIT2 | 555460 | Pharmingen$^{TM}$ BD Biosciences |
